# Beef Cut Classification Using Multispectral Imaging and Machine Learning Method

**DOI:** 10.3389/fnut.2021.755007

**Published:** 2021-10-20

**Authors:** Ang Li, Chenxi Li, Moyang Gao, Si Yang, Rong Liu, Wenliang Chen, Kexin Xu

**Affiliations:** ^1^State Key Laboratory of Precision Measuring Technology and Instruments, Tianjin University, Tianjin, China; ^2^School of Precision Instruments and Optoelectronics Engineering, Tianjin University, Tianjin, China

**Keywords:** multi-spectral imaging, machine learning, beef cuts, classification, feature fusion

## Abstract

Classification of beef cuts is important for the food industry and authentication purposes. Traditional analytical methods are time constraints and incompatible with the modern food industry. Taking advantage of its rapidness and being nondestructive, multispectral imaging (MSI) has been widely applied to obtain a precise characterization of food and agriculture products. This study aims at developing a beef cut classification model using MSI and machine learning classifiers. Beef samples are imaged with a snapshot multi-spectroscopic camera within a range of 500–800 nm. In order to find a more accurate classification model, single- and multiple-modality feature sets are used to develop an accurate classification model with different machine learning-based classifiers, namely, linear discriminant analysis (LDA), support vector machine (SVM), and random forest (RF) algorithms. The results demonstrate that the optimized LDA classifier achieved a prediction accuracy of over 90% with multiple modality feature fusion. By combining machine learning and feature fusion, the other classification models also achieved a satisfying accuracy. Furthermore, this study demonstrates the potential of machine learning and feature fusion method for meat classification by using multiple spectral imaging in future agricultural applications.

## Introduction

Beef plays an important role in daily diet and the food industry because it contains essential nutrients with high biological value (1). The great variability of beef cuts often lead to highly variable qualities, such as tenderness juiciness, and flavor, which are important for consumer's evaluation of beef quality and purchase decision (2). However, the price of high-quality beef cuts, such as sirloin, is much higher than that of low-preference cuts, such as shank and flank. The higher growth demand of beef leads to frauds in retail or supply chain, which means the substitution of high-class beef cuts from low-class. In this sense, concern on beef quality management is highly demanded to identify frauds and prevent any potential hazard. Consequently, beef cut classification becomes essential to meet the demand of consumers and food safety regulators.

Among analytical techniques, the spectroscopy method has been proved to be of great potential for meat analysis because of rapidness, being nondestructive, and minimum preprocessing requirements (3–5). The basic composition of beef, such as water, proteins, myoglobin, fatty acids and lipids, possesses functional groups with certain chemical bonds (O-H, C-H, N-H, etc.) that cause the deviation of a spectrum at a particular wavelength (6). Based on vibrational spectroscopy, the chemometrics method could be applied to extract reliable information from the spectrum and analyze beef cuts qualitatively and quantitatively. However, the spectroscopy approach also suffers from a small sampling area and a lack of spatial information, which limit its application.

The multispectral imaging (MSI) system could capture spectral information at each pixel of a two-dimensional array (7). The three-dimensional hypercube with both spatial and spectral information may potentially make a classification model more detailed (8). Therefore, a high-data dimension also requires feature extraction and fusion to build a reliable classification model (9). In recent years, MSI has been widely applied for quality evaluation and variety classification of food products (10–12). Jiang extracted spectral features and textural features from hyperspectral data of chicken breasts, and then fused them for classification (13). The results demonstrated that fusion features were more conducive to the correct classification of the classifier than single features. Pu also proved that the combination of spectrum and texture information in the modeling process could improve the classification accuracy of different states of pork (14).

Because different types of beef cut might have similar composition, they are difficult to be identified with only spectral information. Meanwhile, the scattering and textural properties of beef are also related to sarcomere length and collagen content. The accuracy of a classification model is dependent on feature extraction, fusion method, and classifier. Feature extraction is recognized as an important approach to obtain an informative feature from high dimensional data. Considering the MIS measurement, feature fusion incorporating both spatial and spectral information has been investigated intensively in a remote sensing area to improve classification accuracy (15). Recently, machine learning (ML) was applied for the processing of spectral and multiple modality data (16–18).

The objective of this study is to develop a classification method by MSI in combination with machine learning-based classifiers. Based on multispectral images of beef cuts, spectral and textural features were extracted to reduce the dimension of data. Three machine learning-based classifiers were used to establish classification models with different feature sets. After the performance of single and multiple-modality features was compared, the best models were put forward. The results confirmed the feasibility of classifying beef cuts with a satisfactory accuracy. Using multiple-modality features, the optimally developed LDA classifier achieved a prediction accuracy of over 90%. Therefore, the proposed approach would provide an effective and empirical reference for meat classification in similar future research and application.

## Materials and Methods

### MSI System and Image Processing

**Figure 1:**
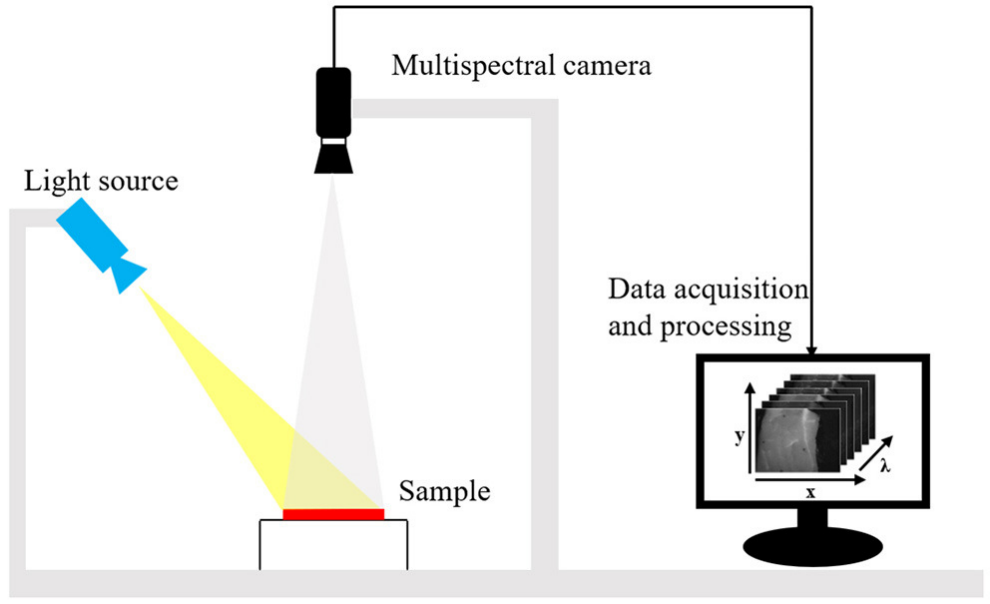
Schematic of the multispectral imaging system.

As shown in Figure 1, the MSI system consists of a light source (HL-2000-FHSA; Ocean Optics, Dunedin, FL, United States) and an adjustable focus lens (Nikon, Tokyo, Japan) coupled with a multi-channel spectroscopic camera (miniCAM5; QHYCCD, China). The spectroscopic camera has one TE-cooled, 16-bit scientific CCD camera and a six-position filter wheel, in which dichroic bandpass filters are mounted. With an adjustable focus lens, the system achieves high-resolution imaging of 1,290 × 960 pixels with six bands, centered at 500, 530, 570, 680, 760, and 808 nm. In this approach, each band covers a relatively wide range of wavelengths (about 16 nm in full-width half-maximum, FWHM), which is typically strong for fast imaging. MSI measurements were performed on fresh beef cuts bought from a local market. The spectra of the beef cuts were also measured with a visible near-infrared (VIS-NIR) spectrometer (BTC611E; BWTEK, United States).

In order to reduce the error caused by the configuration of the MSI system and avoid the oversaturation of the charge-coupled device (CCD) sensor, each image was normalized as a pointwise ratio of dark-noise-corrected intensity from the beef cuts and that from the diffuse reflectance standard (20% reflection; Labsphere Inc., United States), as follows:

(1) $$\begin{array}{r} \begin{array}{c} I_{R}=\;\frac{I_{raw}-I_{dark}}{I_{ref}-I_{dark}}\;\times 20\% \end{array} \end{array}$$

where I_R_ is the calibrated reflectance, *I*_*raw*_ is the raw intensity measured from the test sample, *I*_*dark*_ is the intensity of the dark response, and *I*_*ref*_ is the intensity of the 20% standard reference. This procedure could also compensate inhomogeneous illumination fields and serve as a relative intensity correction.

All the beef cuts were purchased from the Ming Yuan abattoir. The bulls were slaughtered according to standard procedure. The samples were sent to the laboratory within 20 min after purchase, sliced immediately, bagged separately, and stored in a freezing room at −18°C. Before data collection, the samples were thawed in a cold storage room at 6°C for 12 h. The processing steps of all the samples are generally the same, and the time interval of the experiment is short, which guarantees the reliability of the sampling process. To evaluate the representativeness of the samples, emphasis was placed on collecting diverse data with expected variations in color, size, and shape from different sites.

### Classification Model

**Figure 2:**
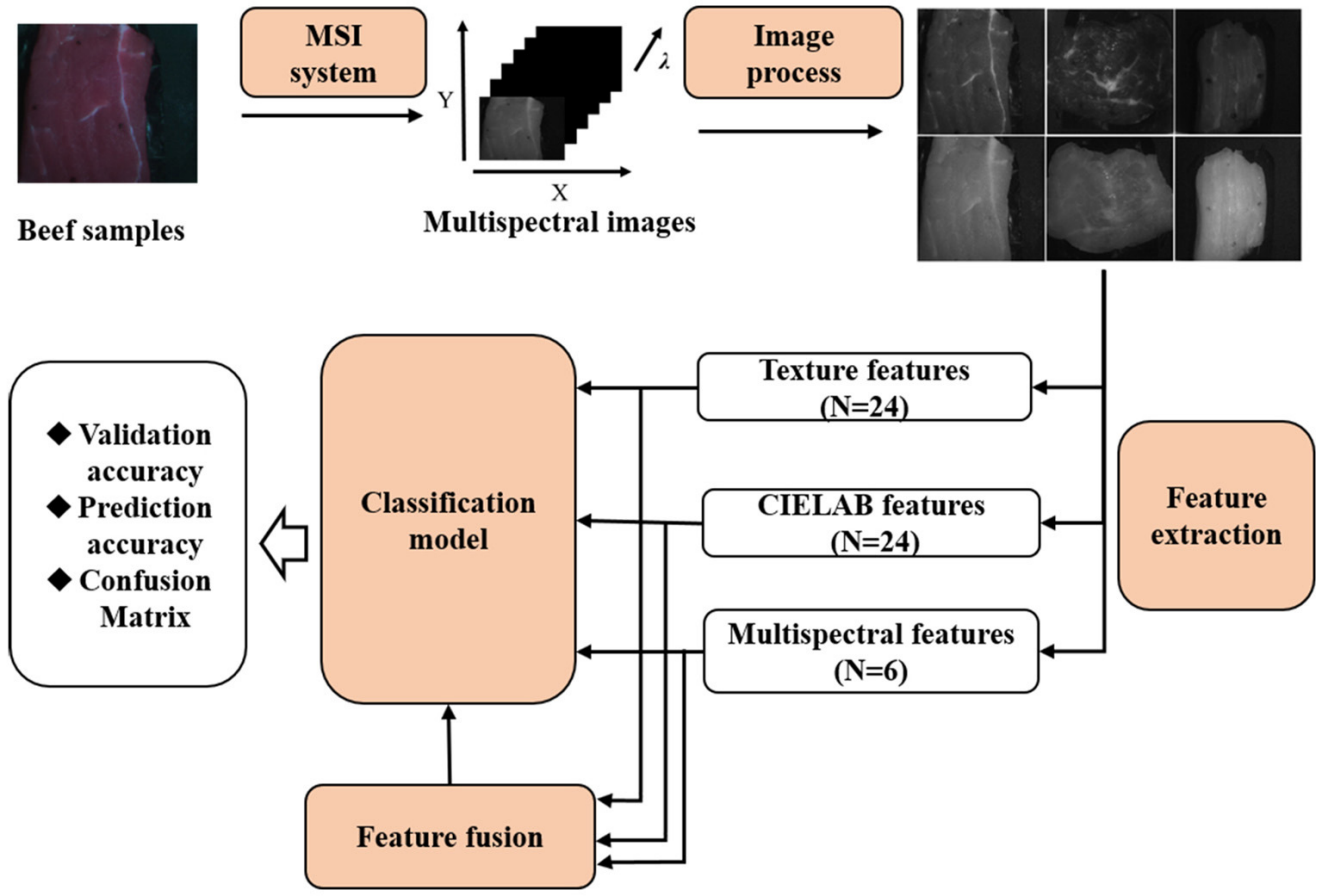
Flow chart of main steps in data acquisition and analysis using multispectral images combined with machine learning for classification of beef cuts.

As shown in Figure 2, the basic steps of the classification model generally involved preprocessing, feature extraction, fusion, and classification. First, the spectral and textual features of multiple-spectral images were extracted and used as single-modality feature sets. After normalization, feature fusion process was introduced by concatenating the spectral and textural features into a single array, which represents the multiple-modality fusion features set. Linear discriminant analysis (LDA), support vector machine (SVM), and random forest (RF) algorithms were applied to establish the classification models based on the single-modality or multiple-modality feature sets. The classification accuracy of different classifier and feature sets were further compared. Details of every step are described in the following sections.

We implemented all the algorithms, i.e., feature extraction, feature fusion, and classifiers, by employing a written script in Python. The built-in function of LDA, SVM, and RF from the sklearn library was used to build the classification models.

#### Feature Extraction and Fusion

The multispectral images contain abundant information, with two spatial dimensions and one spectral dimension, which give more details of the samples. However, the high data curse of dimensionality also makes the classification model more complex. Feature extraction is required to obtain relevant information to make the classification model more accurate and robust.

Considering the anisotropic muscles of beef, the textural features could be extracted with the gray-level co-occurrence matrix (GLCM), which represents the probability that a pixel of specific gray level appears in a specified direction and distance from its neighboring pixels (19). In this study, four statistical textural features were calculated from GLCM, namely, homogeneity, contrast, energy, and correlation, as follows:

(2) $$\begin{array}{r} \begin{array}{c} Homogeneity=\;\overset{N}{\underset{i=1}{\sum }}\overset{N}{\underset{j=1}{\sum }}\frac{g\left(i-j\right)}{1+{\left(i-j\right)}^{2}} \end{array} \end{array}$$

(3) $$\begin{array}{r} \begin{array}{c} Contrast=\;\overset{N}{\underset{i=1}{\sum }}\overset{N}{\underset{j=1}{\sum }}{\left(i-j\right)}^{2}g\left(i,j\right) \end{array} \end{array}$$

(4) $$\begin{array}{r} \begin{array}{c} Energy=\;\overset{N}{\underset{i=1}{\sum }}\overset{N}{\underset{j=1}{\sum }}g\left(i,j\right) \end{array} \end{array}$$

(5) $$\begin{array}{r} \begin{array}{c} Correlation=\frac{\left[\sum_{i=1}^{N}\sum_{j=1}^{N}\left(i-j\right)g\left(i,j\right)-\mu_{x}\mu_{y}\right]}{\sigma_{x}\sigma_{y}} \end{array} \end{array}$$

where $\mu_{x}=\;\sum_{i}\sum_{j}i\cdot g\left(i,j\right){\;,\;\mu }_{y}=\;\sum_{i}\sum_{j}j\cdot g\left(i,j\right)$, $\sigma_{x}=\;\sqrt{\sum_{i}\sum_{j}{\left(i-\mu_{x}\right)}^{2}g\left(i,j\right)}$, $\sigma_{y}=\;\sqrt{\sum_{i}\sum_{j}{\left(i-\mu_{y}\right)}^{2}g\left(i,j\right)}$, and *(i,j)* denote the probability statistics that both pixels with the gray levels *i* and *j* co-occur in the corresponding texture image, and *g(i,j)* is the *(i,j)th* entry in the gray-tone spatial dependence matrix. In general, homogeneity assesses the prevalence of gray-tone transitions; contrast quantifies the local variation in an image; energy reflects the uniformity of the image gray distribution; correlation measures its gray-tone linear dependencies. The four statistical features are obtained from the images of each band and finally formed the textural feature set with 24 variables.

To obtain the spectral features, the multiple-spectral images were quantified into CIELAB color space, and the values in this color space represent the information of the partial band intensity values of the multi-spectrum (20). First, the multispectral images were converted into the CIEXYZ space as follows:


(6) $$\begin{array}{c} \left[\begin{array}{c} X\\ Y\\ Z \end{array}\right]=\;\left[\begin{array}{ccc} 0.412453 & 0.357580 & 0.180423\\ 0.212671 & 0.715160 & 0.072169\\ 0.019334 & 0.119193 & 0.950227 \end{array}\right]\left[\begin{array}{c} R\\ G\\ B \end{array}\right] \end{array}$$

where *R, G*, and *B* are the values of RGB channels and *X, Y*, and *Z* are the values in the CIEXYZ space. Then, the values in the CIEXYZ space were converted into the CIELAB space by the following:


(7) $$\begin{array}{r} \begin{array}{c} L^{*}=116\left(\frac{Y}{Y_{n}}\right)-16 \end{array} \end{array}$$

(8) $$\begin{array}{r} \begin{array}{c} a^{*}=500\left[f\left(\frac{X}{X_{n}}\right)-f\left(\frac{Y}{Y_{n}}\right)\right] \end{array} \end{array}$$

(9) $$\begin{array}{r} \begin{array}{c} b^{*}=200\left[f\left(\frac{Y}{Y_{n}}\right)-f\left(\frac{Z}{Z_{n}}\right)\right] \end{array} \end{array}$$

where:


(10) $$\begin{array}{c} f\left(x\right)=\{\begin{array}{c} x^{\frac{1}{3}}\;,\;x>{\left(\frac{24}{116}\right)}^{3}\\ \left(\frac{841}{108}\right)x+\frac{16}{116},\;\\ x\leq {\left(\frac{24}{116}\right)}^{3} \end{array} \end{array}$$

(11) $$X_{n}=\;\frac{X}{Y},Y_{n}=\;\frac{Y}{Y\;},Z_{n}=\;\frac{Z}{Y}$$

Finally, the L, a^*^, and b^*^ values of the CIELAB space were used as the spectral features.

The spectral and textural features could be used as an input for the classification separately, and are referred to as a single-modality feature set. For multimodal measurement, feature fusion could combine the information obtained by a different modality and generate a composite representation containing more informative description than the single-modality feature could provide. In feature-level fusion, the spectral and textual features could be concatenated integrated into a high-dimensional vector used for classification. In order to eliminate the degradational effect of the feature value, the features were normalized by subtracting the mean and dividing by the standard deviation of each feature vector.

#### Classification Model

In this study, three supervised machine learning-based algorithms, LDA, SVM, and RF, were used to build the classification model for identifying three kinds of beef cuts. Among them, LDA is a generalization of Fisher's linear discriminant, which finds a linear combination of features that characterizes or separates two or more classes. After the input variables are first projected to low dimensions, LDA aims at maximizing the ratio of between-class cluster to within-class cluster. SVM separates different types of samples by mapping the low-dimensional space to the high-dimensional space with a kernel function (21). Generally, the linear kernel function is often a good choice that works with high speed and efficiency. For multi-classification tasks, multiple classifiers can be constructed through “one-vs.-one” strategies to vote. RF generates an ensemble of decision trees and is trained to get majority votes from all the trees with the highest information gain (22). Given an estimate of important variables in the classification, the single tree does not affect the overall prediction. Different trees may show different results from the same classification task, and the most popular result will be selected as the final result.

Considering the dimensionality of the features, we chose the optimization algorithm of “singular value decomposition”, which is good at handling high-dimensional features for LDA. The kernel function and penalty factor were the key parameters of the SVM model. In this study, we used linear as kernel function because of its being highly efficient. The best values for C = 0.6 were optimized using a grid search method. An RF classifier was constructed using the maximum number of features as the square root of the number of features, and the minimum number of samples in the leaf node as 1 to achieve high classification accuracy and efficiency. We tested the results of our proposed model on a various number of trees and found the best performance of RF when the number of trees was 160.

#### Model Evaluation

The experiments were carried out on 550 samples, including 200 cuts of sirloin, 195 cuts of shank, and 160 cuts of flank. All the samples were divided into two subsets manually, namely, calibration set (445 samples) and prediction set (110 samples). The samples in the calibration set were used to establish the classification model. A hierarchical 10-fold cross-validation was performed on the calibration set to verify the performance of the model.

Cross-validation is an effective and widely used technique to evaluate classification models by partitioning the original sample into a training set to train the models, and a test set to evaluate it. In 10-fold cross-validation, the original sample is randomly partitioned into 10 subsamples of equal size. Of the 10 subsamples, a single subsample is retained as the validation data for testing the model, and the remaining nine are used as training data. The cross-validation process is then repeated 10 times (the folds) with every subsample used exactly once as the validation data. For classification problems, the advantage of this method is each fold contains roughly the same proportions of class labels.

The performance of the classification models was evaluated in terms of accuracy, sensitivity, and specificity, which were calculated as follows:

(12) $$\begin{array}{r} \begin{array}{c} Accuracy=\frac{TP\;+\;TN}{TP\;+\;TN+\;FP+\;FN}\times 100\% \end{array} \end{array}$$

(13) $$\begin{array}{r} \begin{array}{c} Precision=\;\frac{TP}{TP\;+\;FN}\times 100\% \end{array} \end{array}$$

(14) $$\begin{array}{r} \begin{array}{c} Recall=\;\frac{TP}{TP\;+\;FN}\times 100\% \end{array} \end{array}$$

where TP is the number of samples correctly classified as positive; TN is the number of samples correctly classified as negative; FP is the number of samples incorrectly classified as positive; FN is the number of samples incorrectly classified as negative. Based on these metrics, confusion matrices were also calculated to explore detailed classification performance.

## Results and Discussion

### VIS-NIR Spectrum and Multispectral Images of Beef Cuts

**Figure 3:**
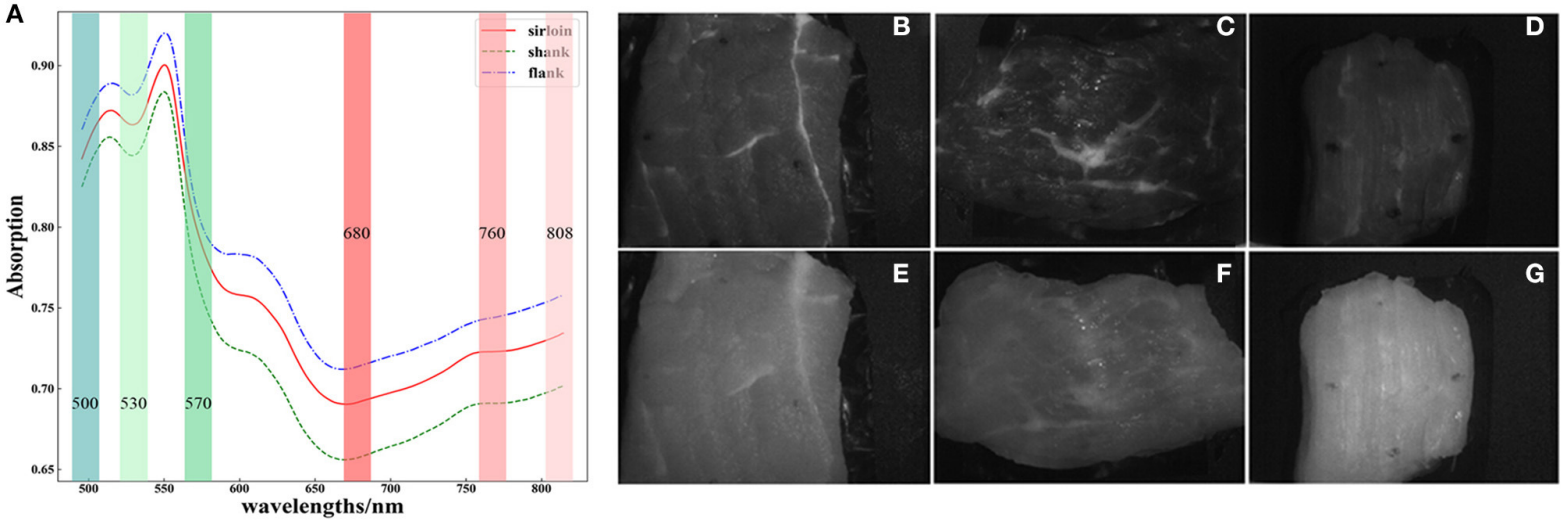
Visible near-infrared (VIS-NIR) spectrum of **(A)** three kinds of beef cut and corresponding multispectral images at 500 nm of **(B)** sirloin, **(C)** flank, **(D)** shank; and multi spectral images at 760 nm of **(E)** sirloin, **(F)** flank, and **(G)** shank.

As shown in Figure 3A, the reflectance spectrum of three types of beef cut shows different intensity and absorption features in the range of 500–800 nm. There are obvious absorption peaks centered at 510, 550, and 640 nm. The absorbance at 510 nm corresponds to light absorption by muscle pigments, while the absorption bands at 550 nm are related to the oxymyoglobin absorption (23, 24). Red meats also show an absorption band at 630 nm, which has been attributed to sulfmyoglobin (24). The weak absorption peak centered at 760 nm corresponds to the third overtone region of O-H or an absorption band produced by deoxymyoglobin (23, 25). Because sirloin contains more water and deoxymyoglobin, the absorption peak around 760 nm is obvious. The main component of flank and shank is a muscle, which contains less water and deoxymyoglobin, resulting in less absorption at 760 nm. These compositions also determine the color and reflectance intensity of the beef, which makes the absorption differ between beef cuts. Among these three kinds of beef cuts, the shank cuts have lower absorption, because they have more white tendons and lipids.

The multispectral images of the beef cuts also show heterogeneity in the arrangement of protein fibers and the presence of intramuscular fat and other tissues, as shown in Figures 3B-G. However, the texture properties also differed with spectral bands. The textural feature also reflects the main components of the beef cuts. Due to the high absorption of myoglobin in muscle, all the three types of beef cuts show lower reflectance at 500 nm, but the heterogeneity of beef was more detailed in this band.

### Results of Classification Model Based on Single-Modality Features

As mentioned above, the three kinds of classification models established were LSVM (linear support vector machine), LDA (linear discriminant analysis), and RF (random forest). In order to prevent overfitting, the regularization parameters need to be set properly in SVM, and it was set as 1 finally. The optimization algorithm in LDA is set as singular value decomposition that is good at handling high-dimensional features. As for RF, the number of trees needs to be set reasonably to achieve high classification accuracy and good computing efficiency. In the attempt, it was found that after the number of trees reached 10, the accuracy of the model no longer improved significantly. The model parameters have been set so far, and model building and prediction are completed using the sklearn library in python3.

As shown in Table 1, the performance of the classification models based on single-modality features was validated by 10-fold cross-validation and prediction test. Generally, classification models based on textural features achieve a better accuracy than those based on spectral features. There was not much difference in the result between two linear classifiers with textual features. The SVM and LDA classifiers achieved a prediction accuracy of 70.91 and 72.73%, respectively. With the spectral features, the RF classifier achieved a cross-validation accuracy of 68.07% and a prediction accuracy of 64.55%. However, the prediction results of the model with spectral features were always lower than that of the model with textural features.

**Table 1:** Performance of the classification models based on single-modality features.

**Types of data**	**No. of Feature Variables**	**Mean Accuracy (%) of Cross-Validation**			**Accuracy (%) of Prediction**		
		**LSVM**	**LDA**	**RF**	**LSVM**	**LDA**	**RF**
MS	6	52.79	66.25	69.25	57.27	43.64	68.18
CIELAB	3	37.51	45.24	46.79	47.27	64.55	50.91
Texture	24	66.46	74.33	71.71	70.91	72.73	59.09

**Figure 4:**
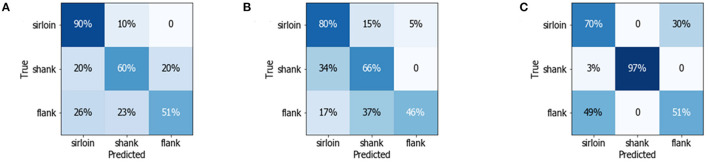
Confusion matrix detailing the multiclass discrimination results of three different beef cuts using **(A)** random forest (RF) model with multispectral data **(B)** linear discriminant analysis (LDA) model with CIELAB data, and **(C)** LDA model with texture data.

The confusion matrix was calculated to present the metrics of sensitivity and recall, providing insights into the classification capability of the models. As shown in Figure 4, the correctly classified results were located on the diagonal, which also indicated the sensitivity of the model. The results demonstrated that most of the sirloin cuts could be correctly classified based on the spectral features. Meanwhile, 97% of the shank could be identified by the LDA classifier with textural features, because the shank cuts have more textural features related to strips of white fascia and unique muscle fiber directions, which can be easily identified as a textual feature. However, the sensitivity of the shank cuts is below 60% with the three classification models, indicating that the flank samples are more difficult to distinguish with single-modality features. According to the confusion matrix, the classification accuracy of different beef cuts varied with the feature set. In that sense, the classification model could be further improved by multiple-modality feature fusion.

### Results of Classification Models With Multiple-Modality Fusion Features

Table 2 summarizes the performance of the classification models with multiple-modality fusion feature sets. The classification accuracy was improved by merging the spectral and textural features. All of the three classifiers performed better with the multi-modality feature fusion set of MS- CIELAB-texture. Regarding feature fusion, the optimally developed LDA classifier shows the biggest improvement, achieving a prediction accuracy of over 90%. The RF classifier is not improved much as the two linear classifiers, because nonlinear classifier needs larger number of samples for training to find good classification criterion.

**Table 2:** Performance of the classification models based on multiple-modality features.

**Types of Data**	**No. of Feature Variables**	**Mean Accuracy (%) of Cross-Validation**			**Accuracy (%) of Prediction**		
		**LSVM**	**LDA**	**RF**	**LSVM**	**LDA**	**RF**
MS + Texture	30	84.25	88.06	75.75	86.36	83.64	80.00
CIELAB + Texture	27	74.83	74.13	71.92	78.18	84.55	61.62
MS +Texture + CIELAB	33	85.37	89.42	80.69	90.00	90.91	80.00

**Figure 5:**
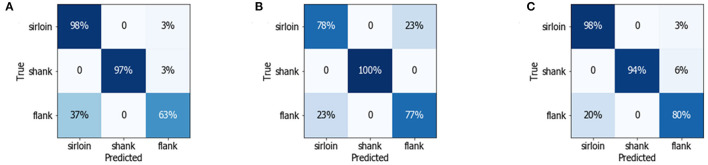
Confusion matrix detailing the multiclass discrimination results of three different beef cuts using **(A)** the linear support vector machine (LSVM) classifier with MS-texture fusion features, **(B)** the LDA classifier with CIELAB-texture fusion features, and **(C)** the LDA classifier with MS-CIELAB-texture fusion features.

As shown in Figure 5, the classification accuracy of the linear classifiers is significantly improved with multiple-modality feature fusion sets. Compared with a single textural feature, the classification accuracy of flank was greatly improved with MS-CIELAB-texture set, achieving a sensitivity of 80% with the LDA classifier, as shown in Figure 5C. Meanwhile, the classification accuracy of sirloin and flank was also improved. The sensitivity of the LDA classifier with MS-textural features set was 98% for sirloin cuts, while the sensitivity for shank also reached an excellent level of 100% with the combination of CIELAB and textural features.

All the results demonstrate that the multiple-modality feature could efficiently improve the accuracy and synergy of the classification model. Thus, feature fusion can combine the sensitive feature by which the classifier established performed better. However, the number of features will also affect the performance of a classifier, such as error tolerance, number of iterations, and training time. More importantly, we should consider all aspects of the model comprehensively when selecting feature set and classification algorithm.

To date, multimodality approaches have shown a considerable capacity to improve the performance of classification and analysis applications. However, most researches have merged redundant information based only on different vibrational spectroscopy techniques. More than improving the classification accuracy, this study tries to provide a better interpretation of a chemometric model and machine vision method.

Recently, portable multispectral devices were developed to meet the increasing requirements of food analysis. This study utilized portable equipment with simplified snapshot measurement, providing the potential to develop a low-cost instrument for online detection. By taking advantage of high-efficiency data acquisition and processing, the MSI approach could be widely applied for many tasks with a low budget. There is also a large potential for combining molecular information and machine vision in data fusion models for applications to different matrices, such as food authenticity and storage.

## Conclusion

In this study, a classification method was proposed to identify different beef cuts with MSI and machine learning-based classifiers. With feature extraction and fusion strategy, multispectral images were converted into single-modality and multiple-modality feature sets and used further as input of machine learning-based classifiers. The results demonstrated that the classification model with multiple-modality fusion feature set performed better than the model with a single feature set. The performance of the LDA classifier was greatly improved with multiple-modality feature fusion, achieving a prediction accuracy of above 90%. This study also provided a better interpretation of the MSI approach, which should be considered for use in routine, nondestructive analyses of the food industry.

## Data Availability Statement

The raw data supporting the conclusions of this article will be made available by the authors, without undue reservation.

## Author Contributions

AL: conceptualization, methodology, software, and writing—original draft. CL: conceptualization, methodology, formal analysis, writing—review and editing, and funding acquisition. MG: software and visualization. RL: validation, formal analysis, resources, and funding acquisition. WC: validation, resources, and funding acquisition. KX: formal analysis, resources, funding acquisition, and project administration. All authors contributed to the article and approved the submitted version.

## Funding

The authors acknowledge the financial support provided by the National Natural Science Foundation of China (Grant Nos: 81871396, 81971657, and 81671727) and the Tianjin Natural Science Foundation (Grant Nos: 19JCYBJC29100 and 20JCZDJC00630).

## Conflict of Interest

The authors declare that the research was conducted in the absence of any commercial or financial relationships that could be construed as a potential conflict of interest.

## Publisher's Note

All claims expressed in this article are solely those of the authors and do not necessarily represent those of their affiliated organizations, or those of the publisher, the editors and the reviewers. Any product that may be evaluated in this article, or claim that may be made by its manufacturer, is not guaranteed or endorsed by the publisher.
